# Efficacy and Safety of Da-Chai-Hu-Tang in Lipid Profiles in High-Risk, Statin-Treated Patients with Residual HyperTG: A 12-Week, Randomized, Active-Control, Open Clinical Study

**DOI:** 10.3390/life12030408

**Published:** 2022-03-11

**Authors:** Young-Shin Lee, Jung-Myung Lee, Hyemoon Chung, Jong-Shin Woo, Byung-Cheol Lee, Weon Kim

**Affiliations:** 1 Department of Internal Medicine, Division of Cardiology, Kyung Hee University Hospital, 26 Kyungheedae-ro, Dongdaemun-gu, Seoul 02447, Korea; leeyoung0831@khu.ac.kr (Y.-S.L.); cardioljm@khu.ac.kr (J.-M.L.); moony@khu.ac.kr (H.C.); imdrwoo@khu.ac.kr (J.-S.W.); 2 Department of Clinical Korean Medicine, Graduate School, Kyung Hee University, 26 Kyungheedae-ro, Dongdaemun-gu, Seoul 02447, Korea; hydrolee@khu.ac.kr

**Keywords:** dyslipidemia, cardiac disease, Da-Chai-Hu-Tang

## Abstract

Da-Chai-Hu-Tang (DCHT) is a herbal extract that has been shown to reduce serum triglyceride (TG) levels in animal experiments as well as small clinical trials. This study aimed to evaluate the efficacy and safety of DCHT in high-risk, statin-treated patients with residual hypertriglyceridemia (hyperTG). This was a 12-week, randomized, active-controlled, open-label, single-center trial. Of these patients, 42 had high cardiovascular risks whose LDL cholesterol levels were controlled by statin treatment; however, with TG levels of 200 to 500 mg/dL they were randomly assigned 1:1 to the OMEGA3 or DCHT group. The primary endpoint was defined as the percentage change in TG at 12 weeks, and changes in other lipid profiles and endothelial cell function were included as secondary endpoints. Safety analyses were also conducted. In the OMEGA3 group, the average TG level decreased from 294.5 ± 72.0 to 210.0 ± 107.8 mg/dL (*p* = 0.004), and in the DCHT group, from 288.7 ± 59.1 to 227.5 ± 98.1 mg/dL (*p* = 0.001). The percentage change in TG was −27.6 ± 33.6 and −22.4 ± 24.1 (*p* = 0.58), respectively, and there was no significant difference between the two groups. There were no severe adverse events in either group. In high-risk, statin-treated patients with residual hyperTG, the administration of OMEGA3 or DCHT for 12 weeks resulted in a significant reduction in TG, and the effect of DCHT was not inferior to that of OMEGA3.

## 1. Introduction

A preprint has previously been published [1].

Dyslipidemia is a major risk factor for well-known cardiovascular diseases (CVD), especially LDL cholesterol [2]. However, previous studies have shown that residual cardiovascular risks persist in patients with optimal LDL cholesterol levels, and other lipid indicators such as triglycerides (TG), non-high-density lipoprotein (non-HDL), and remnant cholesterol have emerged [3,4,5,6,7].

Hypertriglyceridemia (hyperTG) is known as an independent risk factor for coronary artery disease [8,9,10,11,12]. OMEGA3 fatty acid is a drug that has been proven to reduce TG in patients with hyperTG, known through several RCTs for its effectiveness and safety in single-dose or combination with statin treatments [13]. Furthermore, a comparison of icosapent ethyl and placebo identified significant reductions in ischemic events, including cardiovascular death [14]. However, there are no treatments for hyperTG, which have been found to reduce CVD cases in double-blind, randomized studies conducted on patients who are already undergoing statin treatment [15,16]. Therefore, further study will be needed on other drugs that are effective for hyperTG [17].

Several studies have investigated the effects of herbal drugs for dyslipidemia, and Da-Chai-Hu-Tang (DCHT) is one of the insurance-covered herbal extracts in the market. DCHT was found to inhibit hepatic TG biosynthesis in HepG2 human hepatocytes [18]. Additionally, the effect of DCHT has been demonstrated to improve lipid profiles in animal experiments. In hyperlipidemia animal models induced by a high-fat diet, a combined administration of statin, and DCHT for 18 weeks showed a decrease in total cholesterol, liver fat content, and size of adipocytes when compared to the statin-only treated group [19,20,21,22]. In addition, DCHT showed a reduction in lipid profiles in hyperlipidemia patients [23].

To the best of our knowledge, no study has investigated the effectiveness and safety of DCHT compared to OMEGA3 in statin-treated patients. Thus, we designed this study to compare DCHT and OMEGA3 with regard to lipid profile improvement in statin-treated patients at high risk of CVD.

## 2. Materials and Methods

### 2.1. Patients

Adults aged 19 to 70 years with hyperTG (levels greater than 200 mg/dL and less than 500 mg/dL) and with optimal LDL cholesterol levels within the target range through statin treatment for at least 2 months were initially screened [24]. In addition, patients diagnosed with CVD, diabetes mellitus, peripheral artery disease, abdominal aneurysms, or with high cardiovascular risks were enrolled in this trial. The exclusion criteria were as follows: history of an allergic reaction to trial drugs, history of acute coronary syndromes, cerebrovascular disorders, interventional or surgical coronary revascularization within 12 weeks, diagnosis of malignant tumors in the past 5 years, uncontrolled high blood pressure, uncontrolled diabetes, abnormal liver, renal, or thyroid function tests. Those who took drugs that may affect lipid profiles within 8 weeks before participating in the trial were also excluded. Written informed consent was obtained from all patients, and the Ethics Review Board of Kyung Hee University Hospital approved this study (KHUH 2019-07-035-003).

### 2.2. Study Design

**Figure 1:**
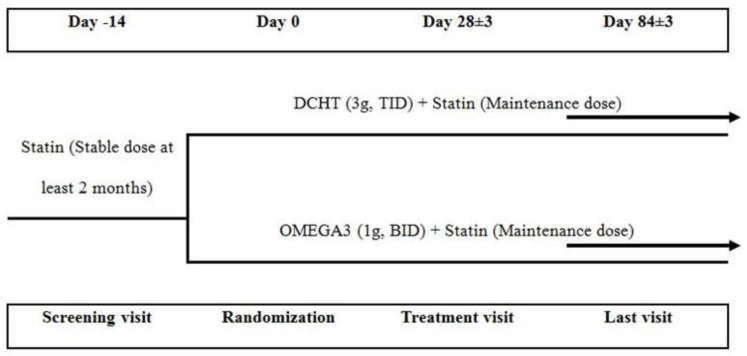
The flow chart of the clinical trial.

The trial was a 12-week, randomized, active-controlled, open-label, investigator-initiated, single-center trial. Since this was a therapeutic exploratory clinical trial without prior clinical studies, we set the sample size to 50 patients. A total of 50 patients were screened from February 2020 to April 2021, at Kyung-Hee Medical Center, Seoul, Republic of Korea, and 42 patients with high cardiovascular risks whose LDL cholesterol levels were stable by statin treatment, but with TG levels of 200 to 500 mg/dL were randomly assigned 1:1 to the OMEGA3 or DCHT groups after a 2-week run-in period. Patients received OMEGA3 1000 mg twice a day or DCHT 1 pack (3 g) diluted in water three times a day for 12 weeks. A flow chart of the clinical trial is presented in Figure 1.

### 2.3. Study Medication

DCHT extract was produced by Hanpoong Pharmaceutical Company (Seoul, Republic of Korea) and comprised of eight herbs as follows: Bupleuri radix, Pinelliae tuber, Zingiberis rhizoma recens, Scutellariae radix, Paeoniae radix, Zizyphi fructus, Ponciri Fructus Immaturus, and Rhei Radix et Rhizoma. The estimated herbs with 10 times the volume of water were mixed and incubated at 90–100 °C for 4 h for extraction. After filtering the extract, the filtrate was sprayed and freeze-dried to obtain 3 g of dried extract. OMEGA3 was used as a 1 g capsule of OMACOR^®^ produced by Kunil-Pharmaceutical Company (Seoul, Republic of Korea). 

### 2.4. Efficacy and Safety Assessment

The primary efficacy endpoint was the percentage change in TG levels from baseline to the end of treatment. The secondary efficacy endpoints included the percentage changes in other lipid profiles, such as non-HDL-C, total cholesterol, LDL-C, HDL-C, Apo A-1, Apo B, and remnant cholesterol at the 12th week from baseline. In addition, changes in endothelial cell function were included as the secondary endpoints. Lipid parameter samples were measured by the laboratories of each hospital using standard procedures. Endothelial cell function was evaluated using the reactive hyperemic index (RHI) using an Endo-PAT 2000 device. The peripheral arterial tone signal was measured from the patient’s fingertip by recording the finger arterial pulsatile volume changes. The results of the 15-min tests were recorded and the RHI score was generated, which represents endothelial cell function. 

Safety analyses were performed by monitoring adverse events, vital signs, laboratory tests including liver and renal function tests, thyroid function test, glycated hemoglobin, and urinalysis. Adverse events were defined as occurring, worsened, or serious during the study period. The causal relationship between the study drugs and adverse events was evaluated.

### 2.5. Statistical Analysis

Continuous variables were expressed as mean (standard deviation (SD)) and compared using the 2-sample *t*-test. Categorical variables were expressed as frequencies and percentages, and the Chi-square test was used for comparison. Two-sided *p*-value < 0.05 were considered statistically significant. The differences in percentage changes in lipid parameters and changes in endothelial cell function tests between the OMEGA3 and DCHT groups were compared using Student’s *t*-test or Mann-Whitney U-test according to normal distribution. The safety assessment variables were analyzed using the *t*-test or the Wilcoxon rank sum test, and the differences in frequencies were compared using Chi-square test and Fisher’s exact test. All analyses were performed using SPSS version 22K (SPSS Korea Inc., Seoul, Korea).

## 3. Results

### 3.1. Baseline Characteristics

Baseline characteristics are summarized in Table 1. The mean age was 63.7 years, and the patient population was predominantly men (69.0%). Demographics were generally balanced between treatment groups. Moreover, there was no significant difference in patients’ past medical history and smoking history between the two groups. The mean baseline TG level was 294.5 ± 72.0 mg/dL in OMEGA3 group and 288.7 ± 59.1 mg/dL in the DCHT group, with no significant difference. There were no significant differences in baseline total cholesterol, LDL, HDL, Apo A-1, non-HDL, remnant cholesterol, and RHI, however the mean Apo B level was slightly higher in the OMEGA3 group (*p* = 0.018) (Table 2).

### 3.2. Efficacy Analyses

In the OMEGA3 group, the average level of TG decreased from 294.5 ± 72.0 mg/dL to 210.0 ± 107.8 mg/dL and the LDL level reduced from 88.6 ± 21.8 mg/dL to 80.8 ± 17.5 mg/dL during the 12-week period. These differences were statistically significant (*p* = 0.004, *p* = 0.049). LDL/HDL cholesterol was significantly reduced (*p* = 0.020), however the increase in HDL cholesterol was not statistically significant (*p* = 0.294). There were no significant differences in other lipid parameters, including total cholesterol, Apo B, non-HDL, TC/HDL, non-HDL, Apo B/Apo A-1, remnant cholesterol, and endothelial cell function indicators. The mean TG level in the DCHT group showed a significant decrease from 288.7 ± 59.1 mg/dL to 227.5 ± 98.1 mg/dL (*p* = 0.001). No significant changes were observed in the other lipid profiles (Table 2). The endothelial cell function, measured with RHI, was the secondary end point of our study, varying from 1.588 to 1.754 in the OMEGA3 group and from 1.901 to 1.858 in the DCHT group. There was no significant difference in the RHI changes between the groups. (*p* = 0.094 and *p* = 0.821, respectively).

**Figure 2:**
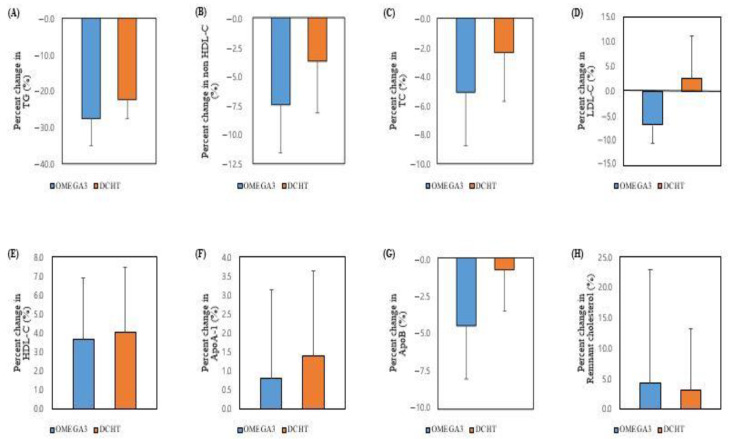
Percent changes in lipid profiles (%): (**A**) Triglyceride; (**B**) Non-high-density lipoprotein cholesterol; (**C**) Total cholesterol; (**D**) Low-density lipoprotein cholesterol; (**E**) High-density lipoprotein cholesterol; (**F**) Apolipoprotein A1; (**G**) Apolipoprotein B; (**H**) Remnant cholesterol. Data are expressed as the percent change (%) ± standard error of the mean (SEM).

Table 2 shows the percentage changes in TGs and other lipid parameters at the end of the treatment. The percentage change of TGs, which was the primary efficacy endpoint in this study, was found to be −27.6 ± 33.6% in the OMEGA3 group and −22.5 ± 24.1% in the DCHT group, with no significant difference (*p* = 0.575). However, there were no significant differences in the percentage change of other lipid profiles, or the secondary outcomes (Figure 2).

### 3.3. Safety Analyses

During the study, the trial treatments were well-tolerated. In the OMEGA3 group, baseline aspartate aminotransferase (AST), alanine transaminase (ALT), and creatinine (Cr) levels were 33.1 U/L, 33.1 U/L, and 0.9 mg/dL, and there were no significant differences (34.2 U/L, 34.5 U/L, and 0.9 mg/dL) at the end of the treatment, respectively. In the DCHT group, the AST, ALT, and Cr levels changed from 32.1 U/L, 35.2 U/L, and 0.8 mg/dL to 34.5 U/L, 35.3 U/L, and 0.8 mg/dL with no statistically significant differences (Table 3).

One patient in the OMEGA3 group and two patients in the DCHT group had adverse events, and the reported adverse events were generally mild. The patient in the OMEGA3 group had subclinical hypothyroidism, which did not require treatment. One patient in the DCHT group had a 3.6-fold increase in ALT levels compared to baseline, and the other patient showed microscopic hematuria on urinalysis. There was no significant difference in the incidence of adverse events between the treatment groups. None of the reported adverse reactions were thought to have a causal relationship with the trial drug.

## 4. Discussion

To the best of our knowledge, this is the first study to evaluate the efficacy of DCHT on lipid profiles compared to OMEGA3 treatment. Our study revealed that the administration of DCHT for 12 weeks led to a significant reduction in TG levels in patients with hyperTG and optimal LDL cholesterol levels by statin treatment. There was no significant difference in efficacy between the OMEGA3 and DCHT treatments. In addition, there were no other significant changes in other lipid profiles or endothelial cell functions.

DCHT is a combination of various natural products used to control hyperlipidemia. The main components are comprised of the following: Bupleurum root, Pinellia tuber, Scutellaria root, Peony root, rhubarb, and Poncirus trifoliate [22]. The effect of the DCHT could be attributed to the pharmacological action of rhubarb [25,26]. The rhubarb inhibits pancreatic lipase, reducing fat absorption within the gastrointestinal tract [25,26]. Previous studies have shown that the administration of rhubarb improves dyslipidemia by inhibiting Acyl-CoA cholesterol acetyltransferase and increasing the expression of cholesterol 7-hydroxylase. These actions lead to an increase in fecal bile acid secretion and lower bile acid pool in the gallbladder [27,28]. Poncirus trifoliata, another component of DCHT, may reduce TG and LDL cholesterol and increase HDL cholesterol through the inhibition of fatty acid synthase and stearoyl-CoA desaturase 1, as well as increase in carnitine palmitoyl transferase 1a and insulin receptor substrate 2 in the liver. It inhibits lipoprotein lipase through regulation of CCAAT-enhancer-binding protein in adipocytes [29]. Beta-sitosterol, induced from Pinellia tuber and Scutellaria root, reduces the absorption of cholesterol in the gastrointestinal tract and increases the expression of the LDL receptor mRNA in hepatocytes [22,30]. Based on the pharmacological actions of the components of DCHT and previous studies, it is expected that DCHT would improve lipid levels in patients with dyslipidemia. In particular, it is believed that the addition of DCHT in statin-treated patients could further increase its effectiveness through the expression of LDL receptors. 

A significant decrease in LDL levels was confirmed in the OMEGA3 group, while no significant changes were observed in the DCHT group for other lipid profiles, including LDL cholesterol. In the DCHT group, the average LDL was 86.2 mg/dL at baseline and 82.9 mg/dL at the end of the treatment. The percentage change in LDL was 2.7%. These results may have been due to differences in the lifestyle of patients including diet, or the lack of cumulative dose of DCHT.

The RHI measured by EndoPAT 2000 showed no significant changes in either group. Previous studies confirmed the effectiveness of OMEGA3 on vascular endothelial function and showed conflicting results depending on the type or total amount of the prescribed OMEGA3 and the target patient group [31,32,33,34]. Based on previous studies, the total cumulative dose of eicosapentaenoic acid and docosahexaenoic acid over 95 g was known to improve endothelial dysfunction, even before significantly decreasing TG levels [35]. However, in diabetic patients, there was no significant improvement in endothelial cells [33]. The results of our study are considered to be due to the inclusion of 12 (60.0%) and 11 (50.0%), respectively, in the OMEGA3 and DCHT groups. Therefore, it is too early to conclude that DCHT is ineffective in improving endothelial cell function, and further research is needed.

Safety issues are an important concern for lipid-lowering agents. The data showed that no serious adverse events occurred in either group. None of the three patients who had side effects complained of any specific symptoms, and the laboratory results improved without any treatment. Considering these results, DCHT dosing is considered to be relatively safe. It is also believed that DCHT could be practically used in the real world due to the benefits of additional TG reduction in statin-treated patients with high cardiovascular risk.

This study has some limitations. First, this study did not perform a double-blind study. Since the effect of OMEGA3 on hyperTG has already been established, we concluded that it is reasonable to design the trial as an active control group in an open-label study. Second, the sample size was relatively small. In addition, long-term follow-up observations are required to confirm that improvements in dyslipidemia lead to a reduction in CVD. Further studies should be performed in a double-blinded manner to minimize bias caused by an open trial.

## 5. Conclusions

In conclusion, in high-risk, statin-treated patients with residual hyperTG, administration of OMEGA3 or DCHT for 12 weeks significantly reduced TG levels, and the effect of DCHT was not inferior to that of OMEGA3.

## Figures and Tables

**Table 1:** Baseline characteristics.

	OMEGA3	DCHT	*p*-Value
Age	62.5 ± 8.6	64.9 ± 8.2	0.36
Gender			0.43
Female	5 (25.0)	8 (36.4)	
Male	15 (75.0)	14 (63.6)	
BMI	26.6 ± 3.5	26.9 ± 2.7	0.74
Past medical history			
Stable angina	5 (25.0)	9 (40.9)	0.28
ACS	8 (40.0)	7 (31.8)	0.58
PCI	11 (55.0)	8 (36.4)	0.23
Stroke	2 (10.0)	2 (9.1)	1.00
Carotid atherosclerosis	5 (25.0)	2 (9.1)	0.23
DM	12 (60.0)	11 (50.0)	0.26
HTN	14 (70.0)	18 (81.8)	0.48
Current smoker	3 (15.0)	2 (9.1)	0.34

BMI: Body mass index; ACS: Acute coronary syndrome; PCI: Percutaneous Coronary Intervention; DM: Diabetes Mellitus; HTN: Hypertension; Data represents the number, frequency, or mean ± SD.

**Table 2:** Changes in lipid profiles before and after the treatment.

	OMEGA3			DCHT			
	Baseline	12 Weeks	Percent Change (%)	Baseline	12 Weeks	Percent Change (%)	*p*-Value
TG	294.5 ± 72.0	210.0 ± 107.8	−27.6 ± 33.6	288.7 ± 59.1	227.5 ± 98.1	−22.5 ± 24.1	0.58
Total cholesterol	151.2 ± 30.8	142.6 ± 26.8	−4.6 ± 14.8	152.2 ± 27.1	146.6 ± 20.0	−2.1 ± 14.2	0.59
LDL-C	88.6 ± 21.8	80.8 ± 17.5	−6.8 ± 16.2	86.2 ± 21.5	82.9 ± 15.4	2.7 ± 40.4	0.49
HDL-C	42.7 ± 8.5	44.2 ± 10.8	3.7 ± 14.5	45.4 ± 8.1	46.7 ± 8.1	4.1 ± 16.0	0.93
Apo A-1	126.9 ± 71.8	128.1 ± 24.9	0.8 ± 10.4	132.8 ± 15.0	133.9 ± 14.9	1.4 ± 10.5	0.86
Apo B	79.5 ± 17.5	74.9 ± 16.5	−4.5 ± 15.8	75.8 ± 12.4	74.6 ± 11.9	−0.8 ± 12.8	0.41
Non-HDL	108.5 ± 30.3	98.0 ± 26.1	−7.5 ± 18.1	106.8 ± 25.5	99.9 ± 20.9	−3.8 ± 20.7	0.54
LDL-C/HDL-C	2.1 ± 0.7	1.9 ± 0.6	−9.0 ± 16.7	1.9 ± 0.5	1.8 ± 0.5	1.4 ± 46.9	0.62
TC/HDL-C	3.6 ± 1.0	3.4 ± 1.0	−6.7 ± 16.6	3.4 ± 0.7	3.2 ± 0.7	−4.1 ± 19.1	0.65
Non-HDL-C/HDL-C	2.6 ± 1.0	2.4 ± 1.0	−9.1 ± 21.9	2.4 ± 0.7	2.2 ± 0.7	−4.8 ± 28.2	0.59
Apo B/Apo A-I	0.6 ± 0.2	0.6 ± 0.2	−4.9 ± 15.0	0.6 ± 0.1	0.6 ± 0.7	−1.3 ± 16.1	0.46
Remnant cholesterol	19.9 ± 10.8	17.2 ± 11.2	4.3 ± 83.4	20.64 ± 9.2	17.00 ± 8.3	3.2 ± 82.1	0.72

TG: Triglyceride; LDL-C: Low-density lipoprotein cholesterol; HDL-C: High-density lipoprotein cholesterol; Apo A-1: Apolipoprotein A1; Apo B: Apolipoprotein B; non-HDL: non-high-density lipoprotein cholesterol; TC: total cholesterol.

**Table 3:** Changes in liver and renal function tests before and after the treatment.

	OMEGA3			DCHT		
	Baseline	12 Weeks	*p*-Value	Baseline	12 Weeks	*p*-Value
AST (U/L)	33.1 ± 13.7	34.2 ± 17.6	0.81	32.1 ± 11.3	34.5 ± 17.3	0.48
ALT (U/L)	33.1 ± 12.5	34.5 ± 18.4	0.96	35.2 ± 14.8	35.3 ± 17.6	0.72
Creatinine (mg/dL)	0.9 ± 0.2	0.9 ± 0.2	0.60	0.8 ± 0.2	0.8 ± 0.2	0.73

AST: aspartate aminotransferase; ALT: alanine transaminase.

## Data Availability

They are available from the corresponding authors upon reasonable request.
